# Psychological Burden and Mental Health Challenges Associated with Occupational Exposure to Zoonotic Pathogens: A Narrative Review and One Health Perspective

**DOI:** 10.3390/tropicalmed11100284

**Published:** 2026-10-09

**Authors:** Carlos Ramiro Silva-Ramos, Saúl Perdomo Cristancho, Yojana Estefany Martínez Hernández, José Ignacio Palacios Osma, Diego Soler-Tovar, Alejandro Ramirez-Hernandez

**Affiliations:** 1 Grupo de Investigación Salud TI_Udistrital, Facultad de Ciencias de la Salud, Universidad Distrital Francisco José de Caldas, Bogotá 110711, Colombia; yemartinezh@udistrital.edu.co (Y.E.M.H.); jpalacios@udistrital.edu.co (J.I.P.O.); 2 Faculty of Nursing, Antonio Nariño University, Bogotá 110231, Colombia; sperdomo22@uan.edu.co; 3 Epidemiology and Public Health Group, Faculty of Agricultural Sciences, Universidad de La Salle, Bogotá 110911, Colombia; diegosoler@unisalle.edu.co (D.S.-T.); alramirez@unisalle.edu.co (A.R.-H.)

**Keywords:** zoonoses, mental health, occupational health, occupational exposure, psychological burden, workforce wellbeing

## Abstract

Zoonotic diseases continue to represent a major global public health challenge, requiring the coordinated efforts of professionals working at the human–animal–environment interface. Although occupational health strategies have traditionally focused on preventing biological hazards and infectious diseases, comparatively little attention has been given to the psychological burden associated with occupational exposure to zoonotic pathogens. Professionals such as veterinarians, animal shelter and rescue personnel, animal health workers, laboratory personnel, researchers, animal control staff, and wildlife conservation professionals are routinely exposed to biological, emotional and ethical challenges that may negatively affect mental health. Important sources of psychological burden include the perceived risk of infection, fear of transmitting pathogens to others, repeated exposure to animal suffering and euthanasia, prolonged outbreak-response activities, occupational uncertainty, and demanding working conditions. These occupational stressors have been associated with adverse mental health outcomes, including burnout, work-related stress, compassion fatigue, anxiety, depression, and sleep disturbances, potentially compromising individual wellbeing, workforce sustainability, and the effectiveness of zoonotic disease surveillance, research, and control. Despite increasing recognition of mental health as a fundamental component of occupational health, important knowledge gaps remain regarding the psychological consequences of occupational exposure to zoonotic pathogens. Greater integration of mental health into occupational health frameworks, together with strengthened research and evidence-based interventions tailored to these professional groups is essential to promote worker wellbeing and support resilient and sustainable systems.

## 1. Introduction

Zoonoses represent a major and growing global health challenge driven by environmental modifications and increasing human–animal–environment interactions [1,2]. The frequent emergence and re-emergence of several zoonotic pathogens (e.g., SARS-CoV-2, vector-borne pathogens) highlight the complex eco-epidemiology underlying zoonotic disease transmission [2,3,4]. These modifications (e.g., climate change, urban expansion, biodiversity loss) have reinforced the need for integrated approaches to disease prevention, surveillance and control [5,6,7].

The One Health approach promotes collaboration across human, animal and environmental health sectors and has contributed to advances in addressing zoonotic threats [5,8,9]. However, One Health discussions have traditionally focused on pathogen dynamics, environmental drivers and disease transmission, while comparatively less attention has been given to the occupational health of the professionals who implement these activities. From a One Health perspective, the health and well-being of this workforce are crucial to the systems responsible for zoonotic disease preparedness and response.

Professionals working with zoonotic pathogens may be exposed to biological hazards while facing substantial occupational, emotional and ethical demands [10,11,12]. Recurrent exposure to potentially infected animals, concerns about pathogen transmission, outbreak-related pressures and emotionally challenging situations (e.g., euthanasia-related decisions), may contribute to psychological and mental health outcomes (e.g., burnout, anxiety, depression) [10,11,13,14]. These potential effects impact not only individual well-being but also workforce sustainability.

Despite growing recognition of mental health as an important component of occupational health, the psychological impact of working with zoonotic pathogens has received comparatively limited attention [11,15,16]. Examining these issues within a One Health framework may therefore help broaden the understanding of zoonotic disease preparedness and response beyond pathogen transmission dynamics to include the wellbeing of the workforce. Therefore, the aim of this narrative review is to discuss the available evidence on mental health outcomes associated with occupational exposure to zoonotic pathogens and to identify relevant challenges and gaps across different occupational settings.

## 2. Literature Search and Selection

A literature search was conducted on 23 July 2026 using PubMed/MEDLINE, Scopus, Embase, Google Scholar and the BVS databases, without a predefined starting date or language restrictions. The search strategy combined concepts related to zoonotic diseases and One Health activities with occupational exposure, mental health outcomes (e.g., burnout, stress, anxiety, depression, sleep disturbances, compassion fatigue), and specific professional groups (e.g., veterinarians, animal handlers, wildlife personnel, laboratory staff, animal control officers). Relevant publications were identified through title and abstract screening, followed by full-text assessment when appropriate. Reference lists of relevant articles were also reviewed to identify additional publications. Selection was based on relevance considering the aim and scope of the review.

## 3. The Importance of Occupational Mental Health

Mental health is increasingly recognized as a fundamental component of occupational wellbeing and an essential determinant of a healthy and sustainable workforce particularly in sectors exposed to biological hazards [17]. Beyond the absence of mental illness, psychological wellbeing encompasses the ability of individuals to meet occupational demands, maintain healthy interpersonal relationships, make effective decisions and adapt to stressful situations and uncertain environments [18,19]. In recent years, occupational health authorities have emphasized the importance of promoting mental health in the workplace, recognizing that psychological wellbeing is closely linked to quality of life, professional fulfillment and overall occupational functioning [20,21]. These concepts align with the World Health Organization’s definition of mental health as a state of well-being that enables individuals to realize their abilities, cope with the normal stresses of life, work productively, and contribute to their communities [22].

The relationship between work and mental health is particularly relevant in occupations characterized by high levels of responsibility, uncertainty, emotional demands and exposure to potentially traumatic events such as biological threats and pathogen handling [23]. In such settings, prolonged exposure to occupational stressors may contribute to psychological distress and reduced job satisfaction. These consequences may affect occupational functioning and the quality of the services provided, including public and animal health responses [24,25]. Therefore, poor mental health may also affect the capacity of organizations to maintain a stable and effective workforce, particularly in sectors that rely on highly trained personnel and rapid decision-making under challenging circumstances or outbreak events [24,25].

Despite growing awareness of the importance of psychological wellbeing in the workplace, occupational health initiatives in zoonotic exposure settings have traditionally focused on preventing physical injuries, accidents and biological hazards, while mental health has often received comparatively less attention [26]. As a result, psychological risks associated with infection concerns and biosafety enforcement have frequently been inadequately monitored and insufficiently addressed within many occupational settings. Although mental health promotion and workplace wellbeing initiatives have received increasing attention in recent years [21,27], important gaps remain regarding the identification and management of occupation-specific psychological stressors. These gaps are particularly relevant in professions where psychological stressors coexist with zoonotic hazards and may accumulate over time [21,26]. Identifying these factors across professional groups and exposure settings is therefore important for developing comprehensive occupational health frameworks that protect workers and support the long-term sustainability of sectors involved in disease prevention and control [20,21].

Job satisfaction may also influence occupational mental health, as higher satisfaction has been associated with greater work engagement and psychological wellbeing [28]. Higher levels of job satisfaction have been associated with lower levels of stress and other psychological disorders among healthcare professionals, including physicians, nurses, laboratory technicians, and other healthcare workers [29]. Nevertheless, occupation-specific conditions, such as long working hours and the handling of hazardous biological agents or high-risk zoonotic diseases, may negatively affect mental health, either temporarily or for as long as these conditions persist.

## 4. Professional Groups Exposed to Zoonotic Pathogens

In the context of the One Health approach, a wide range of professionals work directly or indirectly at the human–animal–environment interface and may be exposed to zoonotic pathogens [15,30]. Although their roles differ, these professionals share occupational contexts characterized by frequent interaction with animals, biological samples, and environments where zoonotic risks are present [15,31] (Table 1).

Veterinarians are among the professionals most frequently exposed to zoonotic pathogens due to their direct contact with animals, tissues, secretions, and other biological materials [32,33,34]. Their professional activities may also involve surveillance, outbreak investigation, and disease control, often requiring close interaction with animals of unknown health status and with pathogens whose zoonotic potential may not be immediately recognized [32,33,34,35]. The frequency of these occupational encounters is illustrated by survey data showing that 16.7% of practicing veterinarians had experienced a zoonotic disease event within a 5-year period [32]. These findings highlight that zoonotic exposure is not an occasional or exceptional component of veterinary practice, but a recurring occupational circumstance that may coexist with other professional demands and contribute to the psychological burden experienced by some workers. However, the presence of occupational exposure does not necessarily imply a uniform psychological response, as the intensity and nature of occupational stressors may vary according to clinical responsibilities, workplace conditions, among others.

Animal shelter and rescue personnel work in environments characterized by high animal density and frequent contact with stray and abandoned animals, often with little or no information regarding their clinical status and vaccination history [36]. Although their work is primarily focused on rehabilitation and adoption, these activities frequently occur under resource-limited conditions and involve the care of animals with untreated illnesses, injuries, or unknown infectious status [36,37]. Daily tasks such as animal handling, feeding, cleaning, transportation, and medical assistance require repeated exposure to biological materials and close contact with animals, creating opportunities for occupational exposure to zoonotic pathogens [15]. These responsibilities may also impose a considerable psychological burden, particularly among personnel involved in euthanasia-related activities [38]. Importantly, the psychological burden in this occupational group may result from multiple interacting factors, including animal suffering, euthanasia, overcrowding, workload, and perceived infection risk, rather than from zoonotic exposure alone. This interaction between emotional and organizational demands is reflected in cross-sectional data from 36 animal shelters, in which employee turnover among personnel involved in euthanasia was positively associated with euthanasia rates [39]. These findings suggest that working conditions surrounding animal care and euthanasia can have measurable consequences for workforce stability and may represent an important component of occupational psychological burden in shelter settings.

Animal-health services act as complex systems that provide healthcare and disease management for animals, primarily food-producing animals, through a combination of public and private sector entities. Traditionally, these services have focused on herd-level disease prevention and control, individual animal clinical care, and food safety inspection [40]. Their role has progressively expanded to include collaboration with public health agencies in the surveillance and control of zoonotic diseases, including integrated surveillance of domestic animals, wildlife, and human populations and collaborative investigation of emerging microbial threats [41]. The workforce delivering these services includes professional veterinarians, paraprofessionals or para-veterinarians, and auxiliaries or community animal health workers, according to their level of formal training [40]. These personnel may encounter emerging and re-emerging zoonotic pathogens originating from domestic or wild animals, creating occupational situations characterized by field exposure, uncertainty, and demanding disease-control activities [41]. The psychological relevance of these conditions is illustrated by a study of field veterinarians involved in a major foot-and-mouth disease containment effort, who reported significantly greater mental than physical distress (mean 3.1 vs. 1.9, *p* < 0.001). Six months after the outbreak, 2.9% of personnel showed indicators of serious mental illness according to the Kessler scale, while vehicle disinfection duties and psychological distress were significantly associated with severe mental illness risk [42]. These findings indicate that intensive animal-health interventions can involve a measurable psychological burden, particularly when occupational demands are intensified by disease-control activities.

Laboratory personnel and researchers, including microbiologists, physicians, and biomedical scientists, play a fundamental role in the detection, characterization, and investigation of zoonotic pathogens [43]. Their work frequently involves the analysis of biological samples obtained from domestic animals, wildlife, vectors, and environmental sources, often in the context of surveillance, outbreak investigations, and research activities [44]. These tasks may require handling biological specimens of unknown infectious status, as well as highly pathogenic microorganisms and newly identified agents whose pathogenic potential remains uncertain [44,45,46]. Consequently, laboratory personnel may encounter both recognized zoonotic pathogens and previously uncharacterized microorganisms, making rigorous biosafety practices, risk assessment, and containment measures essential [44,45]. These occupational conditions may also be accompanied by psychological strain. Among biosafety laboratory personnel, 67.4% reported occupational burnout and 38.9% reported sleep disorders, while those experiencing both conditions had substantially greater odds of impaired work ability (OR = 21.43, 95% CI: 14.30–32.12) [47]. Although these findings demonstrate a substantial burden of psychological and functional outcomes in laboratory settings, they do not by themselves establish that zoonotic pathogen exposure was the specific cause of these outcomes, as biosafety concerns may coexist with other occupational stressors.

Animal control staff are responsible for the capture, transport, and relocation of domestic and wild animals in urban and natural environments. Their work frequently involves direct contact with injured, sick, aggressive, or free-roaming animals whose health status and previous exposure to infectious agents are unknown, as well as with carcasses that may remain infectious after death [48,49]. While domestic animals can serve as important sources of zoonotic pathogens, particularly when stray or free-roaming, interactions with wildlife present additional challenges because of the diversity of microorganisms maintained within natural ecosystems [50,51]. Consequently, animal control personnel may be exposed to known and potentially unrecognized zoonotic agents, making biosafety awareness, appropriate use of personal protective equipment, and field training essential components of their work [51,52]. Beyond infection risk, their occupational environment may involve aggressive animals, unpredictable situations, and emotionally demanding encounters, all of which may contribute to psychological strain. However, compared with other occupational groups, empirical evidence specifically quantifying mental health outcomes among animal control personnel remains limited.

Wildlife conservation personnel, including biologists, ecologists, and other field professionals, conduct much of their work within natural ecosystems. Their activities frequently involve direct or indirect contact with wildlife, arthropod vectors, and environmental sources that may harbor zoonotic pathogens [51,53]. Unlike more controlled occupational settings, fieldwork is often performed in remote areas where exposure to infected animals, vectors, or contaminated environmental sources can be difficult to avoid. Consequently, these professionals may encounter diverse enzootic microorganisms circulating within wildlife populations, many of which remain poorly characterized, while others may be highly virulent [50,51,52,53]. Their position at the interface between wildlife ecosystems and human communities is particularly relevant from a One Health perspective because these activities may place them in settings where pathogen transmission and spillover can occur [54]. Nevertheless, occupational exposure in wildlife settings is highly heterogeneous, and its psychological consequences have received considerably less attention than the epidemiological risks. Multi-year surveys among wildlife professionals illustrate the magnitude of biological exposure, reporting historical bite exposure from rabies reservoir species in 16–42% of participants and rabies pre-exposure prophylaxis rates of 45–53% [52]. These findings demonstrate substantial and potentially preventable occupational exposure in wildlife-related activities, whereas quantitative evidence specifically addressing associated mental health outcomes remains scarce.

## 5. Sources of Psychological Burden Associated with Exposure to Zoonotic Pathogens

Occupational exposure to zoonotic pathogens represents a distinctive occupational risk because it combines biological hazards with uncertainty, ethical dilemmas, and demanding working conditions [10,15,51]. Zoonotic microorganisms include well-characterized pathogens, such as rabies virus, influenza viruses, and pathogenic *Leptospira* spp., as well as newly recognized or poorly characterized agents identified at the interface between natural and urban environments [55,56,57]. For professionals working with these pathogens, the possibility of encountering an infectious agent whose transmission characteristics, clinical consequences, or zoonotic potential are incompletely understood can create an occupational environment in which risk is not always readily predictable [58].

A key source of psychological burden is therefore the perception of infection risk and uncertainty surrounding occupational exposure. A distinction should be made between objective exposure, perceived risk of infection, uncertainty regarding exposure pathways, and confirmed clinical infection. Occupational exposure does not necessarily result in clinical infection; however, the perceived possibility of infection may itself function as a psychosocial stressor, particularly when the circumstances or routes of exposure are uncertain. Even when appropriate biosafety measures are implemented, concerns regarding environmental contamination, effectiveness of preventive strategies, or inadvertent exposure may persist [51,59]. This perceived vulnerability may be particularly pronounced when handling highly infectious biological samples, confirmed zoonotic pathogens, or during outbreak investigations [51,60]. The psychological relevance of this uncertainty is illustrated by evaluations of personnel involved in disease-control operations, in which mental distress was significantly greater than physical distress (mean 3.1 vs. 1.9, *p* < 0.001), and 2.9% of personnel showed indicators of serious mental illness six months after the outbreak [42]. Although these findings do not establish that perceived infection risk was responsible for the observed mental health outcomes, they illustrate how occupational exposure during intensive disease-control activities can occur in a broader context of psychological strain.

A related source of burden is the fear of transmitting zoonotic pathogens to family members, co-workers, or other close contacts [61,62]. This concern extends the perceived consequences of occupational exposure beyond the workplace, as professionals such as healthcare workers that face zoonotic pathogens may worry about inadvertently carrying pathogens into their homes or communities [61,62]. Such concerns may become particularly pronounced during outbreaks involving emerging or poorly understood pathogens, when transmission dynamics remain uncertain and preventive measures may be evolving [62,63]. The potential importance of this concern is illustrated by cross-sectional data from outbreak settings in which 91.6% of frontline healthcare personnel identified fear of transmitting infection to family members as a major stressor, while lack of outbreak-response training was associated with greater odds of severe stress (OR = 2.7, 95% CI: 1.7–4.4, *p* < 0.001) [64]. Although these findings derive from an outbreak setting and cannot be directly generalized to all occupations involving zoonotic exposure, they illustrate how perceived transmission to others can extend occupational risk into personal and family environments and amplify psychological stress.

Another important source of psychological burden arises from repeated exposure to animal suffering, euthanasia, and associated ethical dilemmas. Professionals working with animals may care for individuals with severe disease, poor prognosis, or limited treatment options, creating emotional demands that extend beyond the biological risk of infection [59,65]. Repeated exposure to animal suffering and situations in which considerable professional effort does not result in recovery may contribute to emotional exhaustion [10,65,66]. Euthanasia represents an additional challenge because decisions may involve competing considerations related to animal welfare, professional responsibilities, disease control, and public health [10,67,68,69]. These tensions may be particularly relevant in animal shelters, wildlife management, zoonotic surveillance, and outbreaks in which euthanasia is implemented as a disease-control measure [10,67,68,70]. The organizational dimension of this burden is highlighted by a survey of 305 animal shelter employees across 62 facilities who handle euthanasia tasks. Employees pointed to good leadership from managers as the most important form of help, followed by professional counseling, switching job tasks, and support groups [71]. These findings suggest that the psychological consequences of animal-care activities are influenced not only by the emotional demands of euthanasia itself, but also by the organizational resources available to workers.

Zoonotic outbreaks can further intensify these sources of psychological burden by concentrating multiple occupational stressors over a limited period. Professionals involved in outbreak response may face long working hours, rapidly changing protocols, urgent diagnostic and surveillance activities, and the need to implement control measures despite incomplete information [63,72,73,74]. At the same time, concerns about personal safety and the consequences of delayed or incorrect decisions may increase psychological pressure. Within the One Health framework, these responsibilities may also include communicating zoonotic risks to communities while avoiding unintended consequences such as excessive fear of wildlife or reduced support for conservation activities [75]. Thus, outbreaks can amplify pre-existing occupational demands by combining biological risk, uncertainty, workload, responsibility, and time pressure, potentially creating conditions for sustained psychological strain that may extend beyond the acute outbreak period [63,76]. Overall, the psychological burden associated with work involving zoonotic pathogens appears to arise from the interaction of biological risk with perceived vulnerability, uncertainty, emotional demands, ethical responsibilities, and organizational pressures rather than from exposure alone. However, these factors are not equally relevant across professional groups or exposure settings, and their relative contribution to specific mental health outcomes remains incompletely characterized. This distinction is important because much of the available evidence comes from cross-sectional studies, self-reported symptoms, or outbreak settings, making it difficult to separate the contribution of zoonotic exposure from concurrent factors such as workload, organizational conditions, and exposure to animal suffering (Figure 1).

## 6. Mental Health Outcomes Among Professionals Working with Zoonotic Pathogens

The cumulative effect of occupational demands, biological risk, uncertainty, and emotionally demanding work may be associated with a range of mental health outcomes among professionals working in zoonotic-related settings [77,78]. However, the available evidence is heterogeneous, with substantial variation in professional groups, occupational settings, exposure definitions, outcome measures, and study designs. Most studies are cross-sectional and rely on self-reported symptoms or standardized questionnaires, limiting the ability to determine whether observed psychological outcomes are specifically attributable to zoonotic exposure or to the broader occupational environment. Consequently, the evidence is better interpreted as indicating a mental health burden among professionals working in settings where zoonotic risks are present, rather than establishing a direct causal effect of zoonotic pathogen exposure.

### 6.1. Burnout and Work-Related Stress

Burnout is characterized by emotional exhaustion, mental distancing or cynicism toward work, and reduced professional efficacy resulting from chronic workplace stress [79]. Among professionals working in zoonotic-related settings, potential contributors include persistent perception of biological risk, uncertainty regarding pathogen exposure, heavy workloads, recurrent outbreak response, emotionally demanding situations, ethical dilemmas, and the pressure to balance personal safety with public health responsibilities [10,77,80,81]. Prolonged work-related stress may also affect concentration, decision-making, productivity, and professional commitment, with potential consequences for activities requiring rapid and accurate responses to zoonotic threats [10,25,77].

Evidence from veterinary professionals indicates that burnout is common, although its relationship with zoonotic exposure remains difficult to isolate from other occupational conditions. A systematic review and meta-analysis of 34 studies involving 35,202 veterinary professionals estimated an overall burnout prevalence of 38.68%, with excessive workload and working hours identified among the main occupational stressors [82]. These findings demonstrate a substantial burden of burnout within veterinary practice, but they do not establish zoonotic exposure as its specific cause. Workload, organizational conditions, emotional demands, and exposure to animal suffering may contribute independently or interact with infection-related concerns [77,80,81].

A similar distinction is important when interpreting evidence from high-containment laboratory settings. Among biosafety laboratory personnel, 67.4% reported occupational burnout, and 38.9% reported sleep disorders. Personnel experiencing both conditions had greater odds of impaired work ability (OR = 21.43, 95% CI: 14.30–32.12), while structural equation modeling indicated an indirect relationship between burnout, sleep impairment, and work capacity (β = −0.28, *p* < 0.01) [47]. These findings provide quantitative evidence of substantial occupational strain in a setting involving biological hazards, supporting the relevance of burnout and sleep impairment as interconnected occupational outcomes in high-risk laboratory environments where zoonotic pathogens can be handled.

### 6.2. Compassion Fatigue and Secondary Traumatic Stress

Compassion fatigue refers to psychological distress associated with prolonged exposure to suffering and emotionally demanding caregiving [83]. In animal-related occupations, repeated exposure to sick, injured, abandoned, or dying animals, particularly when treatment options are limited or outcomes are unfavorable, may contribute to compassion fatigue and related forms of secondary traumatic stress [10,66,84]. Veterinarians, shelter personnel, animal rescuers, and wildlife rehabilitation professionals may therefore experience substantial emotional demands as part of routine occupational activities.

Quantitative studies among veterinary and animal-care professionals have reported considerable levels of burnout and secondary traumatic stress, although estimates vary according to the occupational group, population, and measurement approach [66,84]. In a study of clinical veterinary professionals, 36.9% met the study’s criteria for occupational burnout, while 43.1% and 69.8% showed high levels of burnout and secondary traumatic stress, respectively; as well as probable anxiety and depression were identified in 32.6% and 9.0% of participants, respectively [85]. These findings demonstrate the coexistence of multiple psychological outcomes within veterinary populations, but they should not be interpreted as evidence that zoonotic exposure directly produces compassion fatigue. The primary exposure examined in this literature is generally animal suffering and emotionally demanding clinical work, which may coexist with, but is conceptually distinct from, occupational infection risk.

### 6.3. Anxiety and Depression

Anxiety and depression have also been reported among professionals working with animals and infectious disease risks [86,87,88,89,90]. Potential contributors include perceived infection risk, fear of transmitting pathogens to others, uncertainty associated with emerging diseases, high professional responsibility, and chronic occupational stress [88,89]. However, the evidence varies substantially according to occupational context. Studies conducted during infectious disease outbreaks have frequently reported anxiety and depressive symptoms among healthcare and frontline personnel, particularly under conditions of high perceived infection risk and prolonged occupational demands [88,89,90]. These findings are relevant to understanding psychological responses to infectious disease risk but cannot necessarily be extrapolated to routine occupational exposure to zoonotic pathogens, because outbreak conditions introduce additional stressors such as increased workload, rapidly changing protocols, and acute uncertainty.

Evidence from veterinary populations similarly demonstrates substantial psychological burden. Among 1403 veterinary professionals, elevated anxiety, depression, burnout, and secondary traumatic stress were reported relative to general population [91]. However, these findings must also be interpreted in relation to the characteristics of veterinary work more broadly, including workload, exposure to animal suffering, and occupational stressors that are not specific to zoonotic pathogens. Thus, although anxiety and depression are documented among professionals who may encounter zoonotic risks, the available evidence remains insufficient to determine the specific contribution of zoonotic exposure to these outcomes.

### 6.4. Sleep Disturbances

Sleep disturbances may represent both an outcome of occupational psychological stress and a factor that further compromises professional functioning. Chronic work-related stress, long working hours, outbreak-related demands, and concerns regarding occupational infection risk may interfere with sleep quality and recovery [90,92,93]. Sleep impairment can subsequently affect attention, cognitive performance, decision-making, emotional regulation, and overall work capacity [93,94].

Evidence from high-containment laboratory settings provides quantitative support for this relationship. Among biosafety laboratory personnel, 38.9% reported sleep disorders, and the combination of sleep impairment and high occupational burnout was associated with substantially greater odds of impaired work ability (OR = 21.43, 95% CI: 14.30–32.12) [47]. Although these findings do not establish a zoonotic-specific mechanism, they suggest that sleep disturbances may represent an important pathway through which demanding biological-risk environments affect occupational functioning.

Overall, the available literature indicates that professionals working in zoonotic-related environments may experience burnout, work-related stress, compassion fatigue, secondary traumatic stress, anxiety, depression, and sleep disturbances. However, the strength of evidence differs substantially among these outcomes, and few studies directly measure zoonotic exposure alongside standardized mental health outcomes. Much of the current evidence derives from veterinary populations, animal-care professionals, laboratory personnel, or outbreak-response settings in which multiple occupational stressors coexist. Cross-sectional designs, self-reported outcomes, heterogeneous instruments, and inconsistent definitions of exposure further limit causal interpretation. The central evidence gap is therefore not whether psychological burden occurs in these professional groups, but how much of that burden can be specifically attributed to zoonotic exposure after accounting for workload, organizational conditions, animal suffering, outbreak intensity, and other occupational stressors.

## 7. Future Directions

As emerging and re-emerging zoonotic diseases continue to pose significant public health challenges worldwide, greater attention must be paid to the well-being of the professionals responsible for their surveillance, diagnosis, research, and control. Historically, occupational health efforts in these sectors have primarily focused on biological safety and infection prevention, while the psychological consequences associated with working under conditions of uncertainty, recurrent exposure to zoonotic hazards, and demanding public health responsibilities have received comparatively less attention.

Special attention should be paid to occupations that routinely operate at the human–animal–environment interface, where biological, emotional, and ethical stressors frequently coexist and can accumulate over time; future efforts should aim to promote a more comprehensive approach to occupational health for professionals exposed to zoonotic pathogens. This may include greater recognition of psychological risk factors within labor policies, the incorporation of mental health considerations into occupational health programs, and the development of support mechanisms tailored to the realities of these professional groups. Specific recommendations for future studies and policy implementation include evaluating tailored organizational support mechanisms and integrating psychological risk assessments into formal biosafety frameworks.

Evidence from comparable occupational settings indicates that workplace interventions can produce measurable improvements in psychological outcomes. In veterinary teams, a randomized controlled trial of an acceptance and commitment training program reduced stress, burden transfer, and work-related and client-related burnout, with improvements maintained at one-month follow-up [95]. Among healthcare workers, systematic reviews have reported benefits from organizational interventions such as job and task modifications, flexible work arrangements, workload reduction, improved teamwork, and changes in work processes, particularly for reducing burnout and occupational stress [96,97]. These findings suggest that interventions addressing both individual coping skills and modifiable organizational conditions may be relevant for professionals working with zoonotic pathogens, although their effectiveness should be evaluated specifically in zoonotic-related occupational settings.

On the other hand, understanding that this is a phenomenon of public health interest that concerns different sectors, an individualized approach is suggested, comprehending the complexity of the human being and the coping strategies they have built throughout their life and that are useful in the work environment according to their professional role. However, protecting the mental well-being of professionals exposed to zoonotic pathogens should be considered not only as an individual health issue but also as an important component of workforce sustainability.

A better understanding of the factors that contribute most significantly to the psychological burden would facilitate the identification of high-risk populations and support the development of evidence-based policies and specific interventions aimed at improving occupational well-being and the long-term retention of the workforce. Addressing major research gaps requires that future studies should also distinguish between direct occupational exposure to zoonotic pathogens and co-occurring occupational stressors, such as workload, organizational conditions, animal suffering, and outbreak-related pressures.

Finally, additional priority should be given to strengthening research focused on the mental health of professionals exposed to zoonotic pathogens. These efforts can fill important knowledge gaps regarding the occurrence, determinants, and relative impact of different psychological outcomes in professional groups working with zoonotic pathogens in different settings and times. To address these critical research gaps, specific recommendations for future studies include conducting longitudinal and comparative studies using validated instruments and clearly defined exposure measures that would be particularly valuable for determining temporal relationships and identifying factors that may increase or mitigate psychological burden.

## 8. Conclusions

Professionals working with zoonotic pathogens play an essential role in disease surveillance, outbreak response, research and public health response. However, their activities frequently involve a combination of biological risk, uncertainty, emotional demands and ethical challenges that may contribute to a substantial psychological burden. The available literature suggests that professionals working with zoonotic pathogens may experience a range of psychological and mental health outcomes, such as burnout, work-related stress, compassion fatigue, secondary traumatic stress, anxiety, depression and sleep disturbances, although the evidence base remains uneven, heterogeneous, and limited in its ability to establish specific relationships between zoonotic exposure and these outcomes.

Despite increasing recognition of mental health as a fundamental component of occupational health, the evidence regarding the psychological impact of occupational exposure to zoonotic pathogens remains limited and insufficiently characterized and warrants further structured research. Addressing these challenges requires greater integration of mental health within occupational health frameworks, increased research efforts and the implementation of support strategies tailored to the unique demands faced by professionals working with zoonotic pathogens. Protecting the mental wellbeing of these professionals is not only important for individual health but also for maintaining the effectiveness, resilience and sustainability of the workforce responsible for preventing and controlling zoonotic diseases.

## Figures and Tables

**Figure 1:**
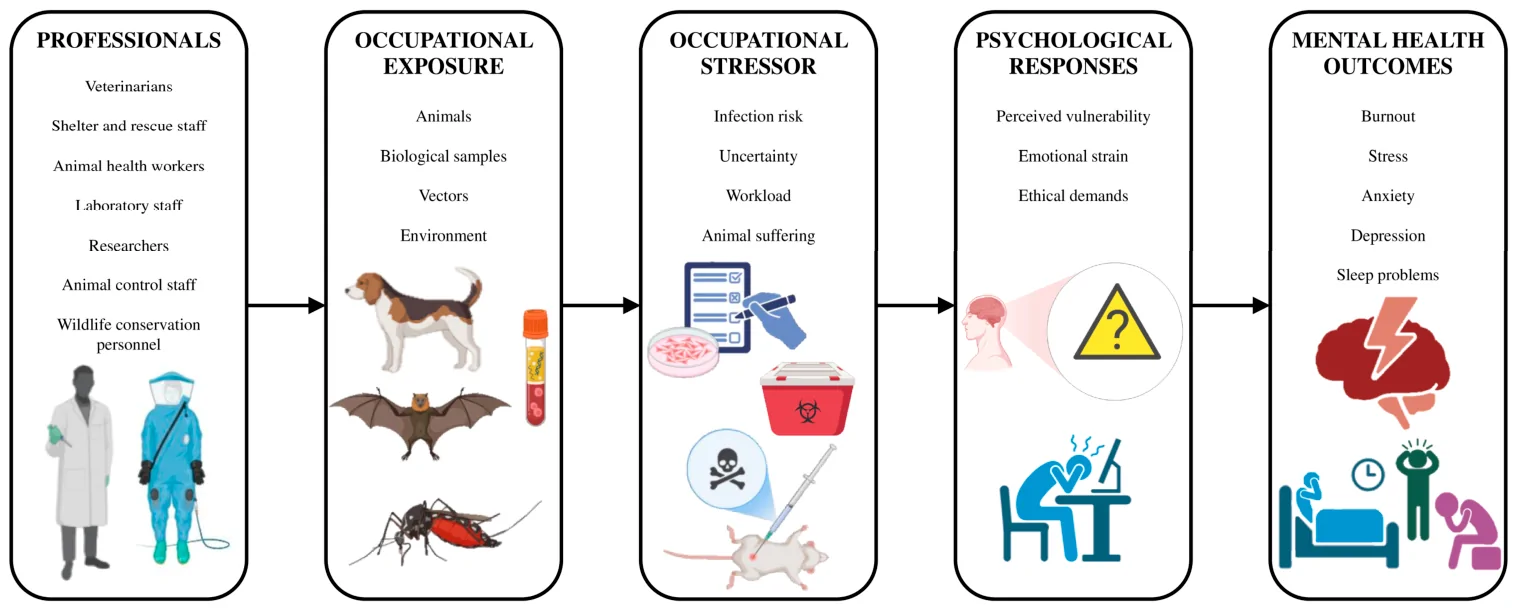
Conceptual framework linking occupational exposure to zoonotic pathogens with psychological responses and mental health outcomes. The framework summarizes the proposed relationship between occupational groups, sources of zoonotic exposure, occupational stressors, psychological responses, and potential mental health outcomes. Created with BioRender.com.

**Table 1:** Occupational groups exposed to zoonotic pathogens and major occupational stressors.

Professional Group	Main Occupational Activities	Sources of Zoonotic Exposure	Main Psychological Stressors	References
Veterinarians	Clinical care, surgery, outbreak response, surveillance	Sick animals, biological fluids, carcasses	Infection risk, euthanasia, ethical dilemmas, workload	[32,33,34,35]
Animal shelter and rescue personnel	Animal care, rehabilitation, adoption, cleaning	Stray animals, unknown health status	Compassion fatigue, euthanasia, overcrowding, emotional burden	[36,37,38,39]
Animal health workers	Herd health, surveillance, field investigations	Livestock, wildlife, biological samples	Long working hours, field conditions, uncertainty	[40,41,42]
Laboratory personnel and researchers	Diagnostic testing, pathogen isolation, research	Clinical specimens, cultures, emerging pathogens	Biosafety concerns, accidental exposure, uncertainty	[43,44,45,46,47]
Animal control staff	Capture, transport, relocation	Wild and domestic animals	Aggressive animals, unknown infectious status	[48,49,50,51,52]
Wildlife conservation personnel	Wildlife monitoring, ecological studies	Wildlife, vectors, environmental samples	Remote work, emerging pathogens, uncertainty	[51,52,53,54]

## Data Availability

No new data were created or analyzed in this study. Data sharing is not applicable to this article.
